# The Yin and Yang of hsa-miR-1244 expression levels during activation of the UPR control cell fate

**DOI:** 10.1186/s12964-024-01967-2

**Published:** 2024-12-02

**Authors:** Paulina Czechowicz, Magdalena Gebert, Sylwia Bartoszewska, Leszek Kalinowski, James F. Collawn, Rafal Bartoszewski

**Affiliations:** 1 Department of Biophysics, Faculty of Biotechnology, University of Wroclaw, F. Joliot-Curie 14a Street, Wroclaw, 50- 383 Poland; 2 Department of Medical Laboratory Diagnostics-Fahrenheit Biobank BBMRI.pl, Medical University of Gdansk, Gdansk, Poland; 3 Department of Inorganic Chemistry, Medical University of Gdansk, Gdansk, Poland; 4 BioTechMed Center, Department of Mechanics of Materials and Structures, Gdansk University of Technology, Gdansk, Poland; 5 Department of Cell, Developmental and Integrative Biology, University of Alabama at Birmingham, Birmingham, USA

**Keywords:** UPR, ER stress, miRNA, microRNA, Cell fate decisions

## Abstract

Regulation of endoplasmic reticulum (ER) homeostasis plays a critical role in maintaining cell survival. When ER stress occurs, a network of three pathways called the unfolded protein response (UPR) is activated to reestablish homeostasis. While it is known that there is cross-talk between these pathways, how this complex network is regulated is not entirely clear. Using human cancer and non-cancer cell lines, two different genome-wide approaches, and two different ER stress models, we searched for miRNAs that were decreased during the UPR and surprisingly found only one, miR-1244, that was found under all these conditions. We also verified that ER-stress related downregulation of miR-1244 expression occurred with 5 different ER stressors and was confirmed in another human cell line (HeLa S3). These analyses demonstrated that the outcome of this reduction during ER stress supported both IRE1 signaling and elevated BIP expression. Further analysis using inhibitors specific for IRE1, ATF6, and PERK also revealed that this miRNA is impacted by all three pathways of the UPR. This is the first example of a complex mechanism by which this miRNA serves as a regulatory check point for all 3 pathways that is switched off during UPR activation. In summary, the results indicate that ER stress reduction of miR-1244 expression contributes to the pro-survival arm of UPR.

**Supplementary Information:**

The online version contains supplementary material available at 10.1186/s12964-024-01967-2.

## Background

The regulation of endoplasmic reticulum (ER) homeostasis is tightly controlled because this organelle is responsible for lipid as well as secretory and membrane protein biogenesis [1, 2]. When the ER equilibrium is disturbed (termed as ER stress), the unfolded protein response (UPR) network is activated to promote cell survival by adequately restoring ER homeostasis and function. When the stress is not mitigated, UPR signaling leads to cell death [1]. To cope with ER stress, the mammalian UPR initializes three main signaling pathways initiated by inositol-requiring enzyme 1 (IRE1), activating transcription factor 6 (ATF6), and protein kinase RNA-like ER kinase (PERK) [3]. IRE1-spliced X-box binding protein 1 (sXBP1) and ATF6 are transcription factors that enhance the expression of ER-resident chaperones and promote ER expansion [3], whereas PERK-mediated phosphorylation of eIF2α inhibits protein synthesis. Although, all of these actions increase the ER folding capacity as well as reduce ER load, they are further supported by IRE1-mediated mRNA and miRNA cleavage and degradation along with ER-associated degradation (ERAD) of the misfolded proteins [3]. Notably, if ER stress remains unmitigated, cell death can occurs through a number of ways including the PERK-related pro-apoptotic transcription factor CCAAT-enhancer-binding protein homologous protein (CHOP) [4], the IRE1- related activation of cJUN NH_2_-terminal kinase (JNK) pathway [5], growth arrest and DNA damage inducible alpha (GADD45A) accumulation [6] and IRE1-dependent decay (RIDD) of mRNA [7, 8], the proteolytic cleavage of ER-associated caspase-4 (in human) [9], and mitochondria-initiated apoptosis that involves the BCL2-binding component 3 (PUMA) and phorbol-12-myristate-13-acetate-induced protein 1 (NOXA) proteins [10].

The ER stress-related cell fate switch is a dynamic process based on changes between the ratios of proadaptive and proapoptotic signals that are being generated by the UPR signaling pathways. In other words, upon ER stress, both the survival and death related actions are being taken, but as the stress duration increases, the equilibrium shifts towards cell death. Thus, although the UPR related cell fate decision accompanies a number of human pathologies including diabetes mellitus, neurodegenerative disorders and respiratory disorders [11], they are still not completely understood and beyond therapeutic control. Given the mutual crosstalk between the UPR signaling pathways, understanding the molecular mechanisms that govern their both proapoptotic and proadaptive signaling pathways is important for determining how cell fate could be therapeutically controlled in cancers for example. Thus, although microRNA (miRNA) are mainly assigned “power switch” functions (reviewed in [12, 13]), their ability to integrate the signals from all arms of UPR to modulate expression of many UPR factors simultaneously makes them potentially the last important step(s) in cell fate-related equilibrium changes.

Here, we report the discovery that hsa-miR-1244 changes during UPR signaling fine tune this pathway to modulate cell fate decisions.

## Materials and methods

### Cell lines and culture conditions

HeLa S3 and Calu3 cells were obtained from ATCC (CCL-2.2™ and HTB-55). 16HBE14o- cells were obtained as previously described [14]. Cells were cultured in Minimum Essential Modified Eagle’s Medium (Invitrogen) with 2mM l-glutamine (Sigma-Aldrich), antibiotics (100 units/mL of penicillin, 100 µg/mL of streptomycin (Sigma-Aldrich)) and 10% fetal bovine serum in a humidified incubator at 37 °C in 5% CO_2_ in 6-well plates and allowed to grow to 70–80% confluence prior to the start of the experiments.

### Induction of ER stress and activation of the UPR

Pharmacological induction of ER stress and activation of the UPR were performed according to previously described methods [15]. Briefly, cells were treated with ALLN (calpain inhibitor I), 100 µM, Abcam, ALLN DMSO solution (ab146608), Tunicamycin (Tm; 2.5 µg/ml, Sigma, T7765), Thapsigargin (Tg, 50 nM, Sigma, T9033), Brefeldin A (BA, 0.4 µg/ml, Sigma, B6542), or Dithiothreitol (DTT, 4 mM, Sigma 43815) for the time periods specified. Cells were also treated with mentioned stressors in the presence of pharmacological inhibitors specific for each UPR branch: 4µ8C (IRE1α inhibitor in 50µM final concentration; Sigma SML0949), Ceapin-A7 (ATF6 inhibitor in 4µM final concentration, Sigma SML0843) and ISRIB (PERK inhibitor in 1µM final concentration; Sigma SML0843). Triazoloacridone 5-{[3-(Dimethylamino)propyl]amino}-8-hydroxy-6 H-[1,2,3]triazolo[4,5,1-de]acridin-6 one (C_18_H_19_N_5_O_2_, M_W_ = 337.38 g/mol) (compound C-1305) was synthesized as previously described in [16]. Prior to the experiments, the compound was freshly dissolved in DMSO as a 3 mM stock solution.

### miRNA analogs transfections

Cells were seeded onto 6-well plates or 35 mm dishes and transfected at 70–80% confluence with Lipofectamine RNAiMax (Thermo Fisher Scientific) according to the manufacturer’s protocol. mirVana miRNA mimics and mirVana miRNA Inhibitors (Thermo Fisher Scientific) were used at final concentrations of 10 and 150 nM, respectively. mirVana mimics and inhibitors used in this study: miR-1244 mimic (Assay ID: MC13172) and inhibitor (Assay ID: MH13172). In all experiments, cel-miR-67 was used as a scramble control since it has no homology to any known mammalian miRNA (Assay ID: MC22484) [17]. As an additional control, Ambion siRNA Negative Control 1 no. 4,390,843 was used as well. The degree of miRNA overexpression or knockdown was determined by qRT-PCR. Following the transfection, cells were cultured for 48 h prior to analysis.

### Isolation of RNA and small ncRNA

Total RNA containing the small ncRNA fraction was isolated using miRNeasy kit (Qiagen, 217004). RNA concentrations were calculated based on the absorbance at 260 nm. RNA samples were stored at -70 °C until use. The RNA quality was verified with Agilent 4200 Tape Station system.

### Genome wide mRNA and miRNA analysis and bioinformatic tools

The genome wide mRNA and miRNA analyses were performed as we previously described in [18]. The volcano plots were analyzed with the VolcaNoseR and Manhattan distance methods [19]. The Venn diagrams were prepared with InteractiVenn [20]. The gene ontology analysis was performed with Enrichr web server [21], whereas MIRDIP web server was used to identify miR-1244 targets [22]. The heat maps and hierarchical clustering were performed with the Morpheus web server (https://software.broadinstitute.org/morpheus).

### Measurement of miRNA and mRNA levels using quantitative real time PCR (qRT-PCR)

We used TaqManOne-Step RT-PCR Master MixReagents (Applied Biosystems) as described previously [15] using the manufacturer’s protocol. The relative expressions were calculated using the comparative relative standard curve method [23]. We used glyceraldehyde-3-phosphate dehydrogenase (*GAPDH*) mRNA and small nucleolar RNA, C/D Box 48 (*RNU48*) *RNA*, as the relative controls for our studies. The relative control stability during experiments was verified with *18 S* rRNA and *TATA-box Binding Protein* (*TBP*). TaqMan probes ids used were: *GAPDH* - Hs03929097_g1; *18 S* - Hs03003631_g1; *TBP* - Hs00427620_m1; *BIP* (*HSPA5*)- Hs00607129_gH; *CHOP* (*DDIT3)*- Hs00358796_g1; *DNAJC3* - Hs00939346_m1; *PUMA* (*BBC3*) - Hs00248075_m1; *NOXA (PMAIP1)* - Hs00560402_m1; *TP53* - Hs01034249_m1; *XBP1s* - Hs03929085_g1; *XBP1u* - Hs02856596_m1; *XBP1* - Hs00231936_m1; *SRP54A* - Hs0128080_m1; *SEC23B* - Hs00197211_m1; *DNAJB9* - Hs01052402_m1; *EDEM1* - Hs00976004_m1; *ERN1* - Hs00176385_m1; *PERK (EIF2AK3)* - Hs00984003_m1; *ATF6* - Hs00232586_m1; *MAGT1* - Hs00259564_m1; *HSPD1* - Hs01036753_g1; *XIAP* - Hs00745222_s1; *RNU48–00106*; hsa-miR-1244–002791.

### Monitoring caspase 3 and caspase 7 activity

Because caspase 3 and caspase 7 have the same peptide recognition motif and thus several endogenous protein substrates, the activity of these caspases is redundant [24]. Although our main goal was to measure caspase 3 activity, the commercial assays do not distinguish between these two cysteine proteases. Therefore, we applied the caspase-Glo 3/7 assay (Promega, Madison, WI, USA) to measure relative caspase activity as described previously [6]. Briefly, cells the day after transfection with the specified miRNA analogs were seeded onto 96-well luminescence assay white plates with clear bottoms (Corning Inc., 3903, Corning, NY, USA). The next day, cells were treated with ER stressors or vehicle (1% DMSO) for the indicated time points. Following treatment, cells were washed with PBS and the Caspase-Glo 3/7 assay (Promega) was performed in accordance with the manufacturer’s instructions using the GloMax-Explorer Detection System (Promega). The results were normalized to the values obtained from the vehicle CTRL treatments.

### Real-time cell viability assay

For real-time monitoring of cell viability, we applied real-time and label-free holographic microscopy-based monitoring of cell death and viability using HoloMonitor M4^®^ time-lapse cytometer (Phase Holographic Imaging PHI AB, Lund, Sweden). Holographic microscopy was used to follow the optical thickness and irregularity of cells exposed for up to 24 h to Tm or Tg in the presence or absence of miR-1244 mimic or antagomiR. The images from up to 8 independent optical fields were collected and analyzed according to manufactures instructions with HoloMonitor^®^ App Suite software. Healthy cells are irregular in shape and thin, whereas dying cells are round and thick [25–28]. For all analysis, the same cells parameters qualification was applied.

### Statistical analysis

Results were expressed as means ± standard deviations (SD). Statistical significance among means was determined using the Student’s t-test (two samples, paired and unpaired) as well as two-way ANOVA test with *P* < 0.05 considered significant.

## Results

**Fig. 1:**
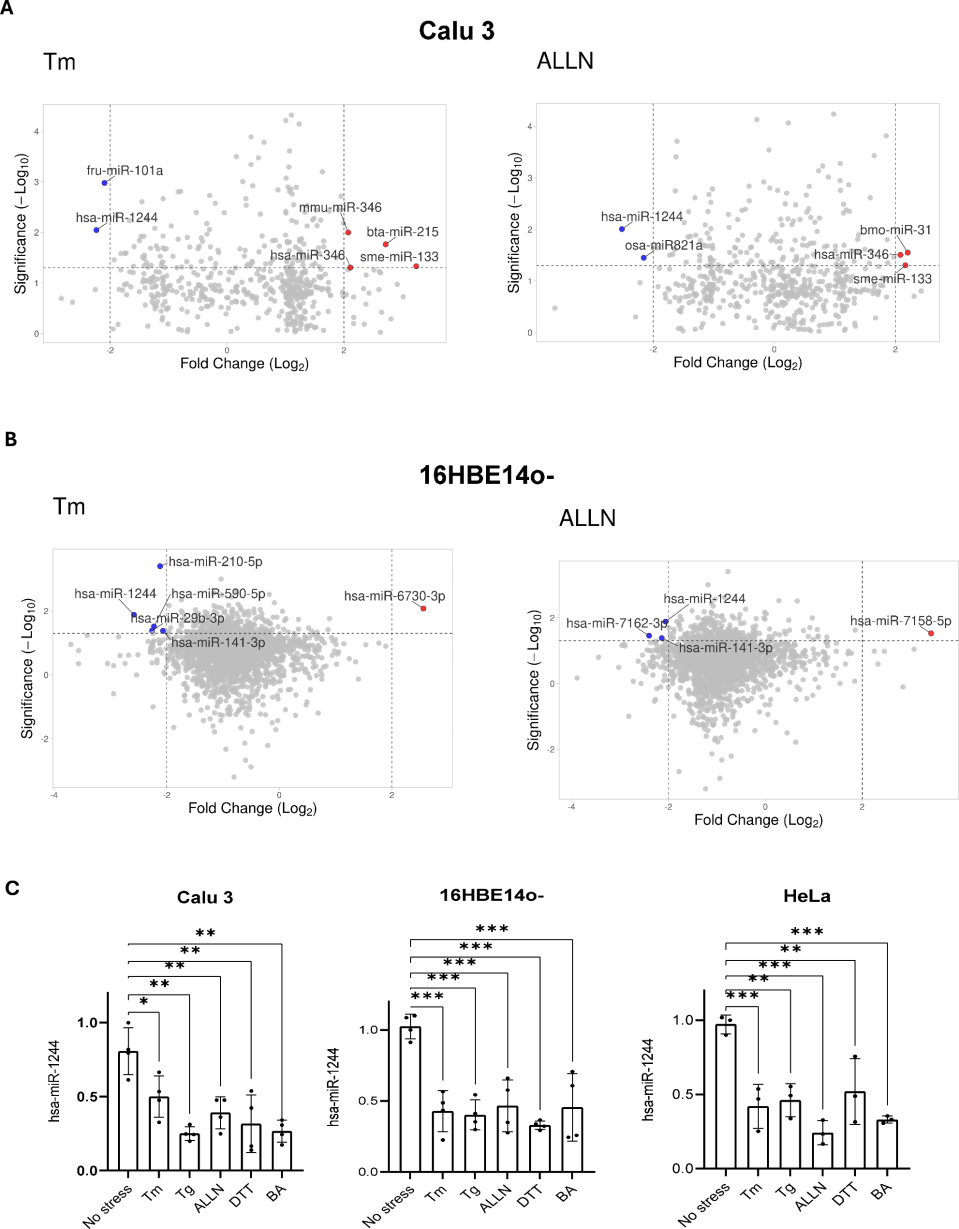
UPR is accompanied by reduced levels of miR-1244. (**A**). Volcano plots illustrate the ER stress related changes in miRNA expression profiles in Calu 3 cells exposed to Tm (2.5 µg/ml) (left) and ALLN (100µM) (right). The miRNAs that were changed by 2 log_2_ fold (P value < 0.05) were selected, and downregulated ones are marked with blue and upregulated ones are marked with red. (**B**). Volcano plots are shown that represent ER stress-related changes in miRNA expression profiles in 16HBE14o- exposed to Tm (left) and ALLN (right). The miRNAs that were (P value < 0.05) changed by 2 log_2_ fold (P value < 0.05) were selected, and downregulated ones are marked with blue whereas upregulated ones are marked with red. (**C**) Relative miR-1244 expression in different cell types following UPR activation with various ER stressors. Calu-3, 16HBE14o-, and HeLa cells were treated with different ER stressors (Tm, 2.5 µg/ml Tg – thapsigargin, 50 nM, ALLN 100µM, dithiothreitol – DTT 4 mM, and brefeldin A – BA, 0.4 µg/ml) for 8 h and total RNA enriched in miRNAs was isolated. mir-1244 levels were quantified by qPCR and normalized to RNU48. Results are plotted as fold changes over control miR-1244 levels in untreated cells. Data represents the mean ± SD of three independent experiments. *P < 0.05, **P < 0.001, ***P < 0.0001 were considered significant

To identify miRNAs that are reduced upon ER stress in both cancer and noncancer human airway epithelial cells (Calu 3 and 16HBE14o-, respectively), we compared miRNA expression profiles obtained with two independent genome-wide approaches, genome-wide miRNA expression arrays (for Calu 3 cells) [15] and next generation sequencing (for 16HBE14o- cells) [18]. Both of these approaches were based on two classic ER stress models, a proteasome inhibitor, ALLN (calpain inhibitor I) and a glycosylation inhibitor, tunicamycin, to activate the UPR. The activation of both UPR adaptive and apoptotic signaling was verified by parallel genome-wide mRNA expression arrays (for Calu 3 cells) [15] and next generation sequencing (for 16HBE14o- cells) [6]. The increase in heat shock protein family A (Hsp70) member 5 (*HSPA5*, also known as *BIP*) mRNA levels was accompanied by a similar elevation of *CHOP* (*DDIT3*) mRNA levels in both cell lines and in both ER stress models, confirming that the UPR was fully activated (Supplemental Fig. 1) We selected miRNAs that were decreased by both ER stressors in both Calu 3 and 16HBE14o- cells (Fig. 1AB). The only miRNA found under all these conditions was miR-1244 (Fig. 1AB). Next, we verified that ER-stress related downregulation of miR-1244 expression was independent of the pharmacological ER stressor as well as that this miRNA was also reduced in other human cells such as HeLa S3. As shown in Fig. 1C, hsa-miR-1244 was reduced independently of the mechanism of UPR induction not only in Calu 3 and 16HBE14o- cells, but also in HeLa cells. Furthermore, miR-1244 levels remained unaffected when the cells were treated with the cytotoxic agent C-1305 (Supplemental Fig. 2). Taken together, this data suggests that the downregulation of miR-1244 during ER stress is representative and potentially important for the UPR in human cells. However, the magnitude of hsa-miR-1244 reduction in response to ER stress varied among cell types and ER stressors and this may reflect differences in basal expression levels of hsa-miR-1244.

**Fig. 2:**
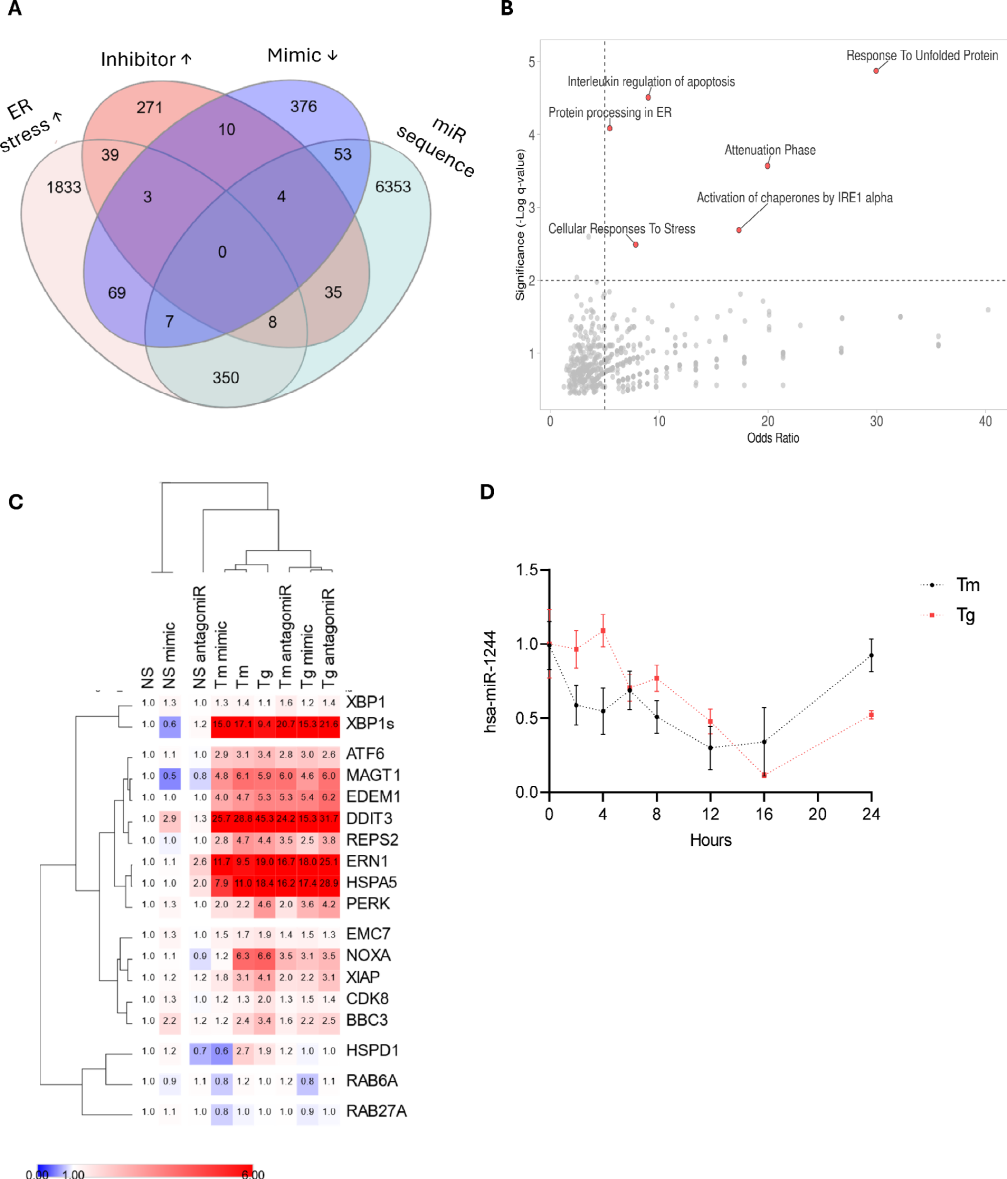
miR-1244 targets are involved in UPR. (**A**) Venn diagram summarizing the strategy used to select ER stress related miR-1244 targets. The analysis included mRNAs that were either downregulated by miR-1244 mimic or upregulated by its inhibitor (by 1.5 log_2_ fold). ER stress upregulated genes in 16HBE14o- cells (by 1.5 log_2_ fold) and the genes that were predicted with MIRDIP to contain miR-1244 binding sites are shown (with P value < 0.05). (**B**) The summary of the gene ontology analysis of selected miR-1244 targets − (104 mRNAs) that were upregulated by ER stress and upregulated by inhibitor or downregulated by mimic are shown (Supplemental Data Set). (**C**) The hierarchical clustering analysis (Pearson correlation based) of qPCR analyzed changes in expression of miR-1244 UPR related targets in HeLa cells exposed to Tm or Tg for 16 h and transfected with scramble control, miR-1244 mimic or antagomiR are shown. The values represent the mean of 3 independent experiments. (**D**) HeLa cells were treated with Tm (2.5 µg/ml) or Tg (500nM) for the times indicated, and total RNA enriched in miRNAs was isolated. MiR-1244 levels were quantified by qPCR and normalized to *RNU48.* Data represents the mean ± SD of three independent experiments. *P < 0.05 was considered significant

Thus, to define the capacity of miR-1244 regulatory activity, we performed an NGS based genome-wide analysis of mRNA expression profiles in HeLa cells after miR-1244 overexpression with its synthetic analog (hsa-miR-1244 mimic) and more importantly, after inhibition of endogenous miR-1244 with a specific inhibitor (hsa-miR-1244 antagomiR). Here we applied a miR-1244 target selection analysis that would allow us to focus on this molecule’s role in UPR signaling. Given that miRNA effects on gene expression serve a modulatory function and result from simultaneous interactions of many different miRNA with specific targets [29], we set our selection threshold to 1.5 log2 fold change. Furthermore, since ER stress reduced miR-1244 levels, we decided to include in our analysis transcripts that were identified to be upregulated during ER stress in our previous genome wide analysis in 16HBE14o- cells [6]. As shown in Fig. 2A, this approach resulted in identification of a large group of potential direct miR-1244 target mRNAs (107 mRNAs) (Supplemental Data Set D) that were upregulated by ER stress and upregulated by inhibitor or downregulated by mimic. The functional aspects of these gene changes were strongly and significantly related to UPR that included IRE1 signaling, protein processing in ER, and interleukin-regulated apoptosis (Fig. 2B, Supplemental Data Set E). Notably, the potential miR-1244 targets included crucial receptors and mediators of UPR including *XBP1*, *ATF6*, *IRE1* (*ERN1*) and PERK as well as genes implicated in ER stress related apoptosis such as *NOXA*, *PUMA* (*BBC3*) or *XIAP* (Fig. 2C).

Next, to verify these changes in the UPR, our follow up studies were based on the independent validation (qPCR) of these potential miR-1244 targets, as well as the related changes in *BIP* and *CHOP* expression in HeLa cells exposed to ER stress and transfected with either miR-1244 mimic or inhibitor. All these genes were tested in no stress conditions as well. Since ALLN is known to partially inhibit IRE1 activity, as a secondary ER stress model we used thapsigargin (Tg), an inhibitor of sarcoplasmic/endoplasmic reticulum Ca^2+^ ATPase [30]. As shown in Fig. 2D, both Tm and Tg had similar effects on miR-1244 levels and reduced this miRNA expression significantly from 8 to 16 h of ER stress exposure. Notably, as shown by hierarchical clustering analysis, reduction of miR-1244 in no ER stress condition by its antagomiR supports expression of prosurvival factors including *IRE1*, *XBP1s*, and *BIP* and therefore resembles proadaptive UPR activity. Importantly, in no stress conditions, miR-1244 overexpression increases the levels of *CHOP (DDIT3)* and *BBC3*, and thus promotes apoptotic signals (Fig. 2C). Taken together, these data suggest that the downregulation of miR-1244 during UPR supports the activities that restore ER homeostasis and assure survival.

Notably, however, none of the miR-1244 UPR related potential targets had the clearly bidirectional pattern (decrease/increase expression) upon mimic and inhibitor (antagomiR) treatments, respectively, in all 3 experimental settings (Supplemental Fig. 3). Both *ERN1* and *XBP1s* contain putative miR-1244 biding sites, however, miRNA overexpression had no significant effect on these two transcripts, whereas miR-1244 antagomiR rescued *XBP1s* mRNA only during Tg treatments (Supplemental Fig. 3A). Notably, however, the inhibition of miR-1244 resulted in accumulation of *ERN1* transcript in both ER stress models (Supplemental Fig. 3B). As shown in Supplemental Fig. 3CD, miR-1244 overexpression had a mild reducing effect on both *PERK* and *ATF6* mRNA levels in Tg treated cells only, while antagomiR was ineffective in all conditions. A similar pattern has been observed by magnesium transporter 1 (*MAGT1*), where miR-1244 overexpression related downregulation was observed in all conditions, while antagomiR remained ineffective (Supplemental Fig. 3E). Mild effects of both mimic and antagomiR during ER stress were also observed for ER degradation enhancing alpha-mannosidase like protein 1 (*EDEM1*) (Supplemental Fig. 3F). The mRNA of this potential target of miR-1244, was slightly elevated by antagomiR in both Tm and Tg models but reduced by mimic only after Tm. Interestingly, both miR-1244 inhibition and overexpression resulted in reduced heat shock protein family D (Hsp60) member 1 (*HSPD1*) levels during ER stress (Supplemental Fig. 3G). Importantly, despite the fact that BIP transcript lacks mir-1244 binding site, the levels of this mRNA were significantly induced in the presence of miR-1244 inhibitor in ER stress conditions (Tm and Tg), while mimic had only mild reducing effect on this transcript during Tm treatment (Supplemental Fig. 3H). Taken together, this data suggests that reduction of miR-1244 during ER stress supports IRE1 signaling as well as BIP activity.

In contrast, this miRNA could provide a link to the mitochondrial UPR, since both overexpression and inhibition of miR-1244 reduced the expression of a crucial mitochondrial chaperone, *HSPD1* [31]. The link between miR-1244 and mitochondrial homeostasis is also reflected in the expression profiles of its two other potential targets, *PUMA* (*BBC3*) and *NOXA* (*PMAIP1*), that link the UPR with mitochondrial apoptosis [32]. As shown in Supplemental Fig. 4A, miR-1244 overexpression elevates the *BBC*3 levels in no stress conditions, whereas during Tm and Tg treatments, both mimic and antagomiR significantly attenuated the levels of this transcript. miR-1244 related effects were also observed for *NOXA* (Supplemental Fig. 4B). Furthermore, deregulation of miR-1244 expression during ER stress, resulted in significantly lower levels of another potential direct target of this miRNA – antiapoptotic *XIAP*, that is also responsible for controlling caspase activity (Supplemental Fig. 4C). Despite the fact that miR-1244 overexpression was indirectly elevating *CHOP* levels under control conditions, it was not effective in Tm treated cells, whereas during Tg treatments, mimic transfection also resulted in reduced levels of this transcript that were similar to antagomiR (Supplemental Fig. 4D). Although this data does not pinpoint which of the cell death pathways involved transcripts is a crucial target of miR-1244, they suggest that maintaining lower levels of this miRNA during ER stress may be crucial for maintaining survival for a longer period of time.

**Fig. 3:**
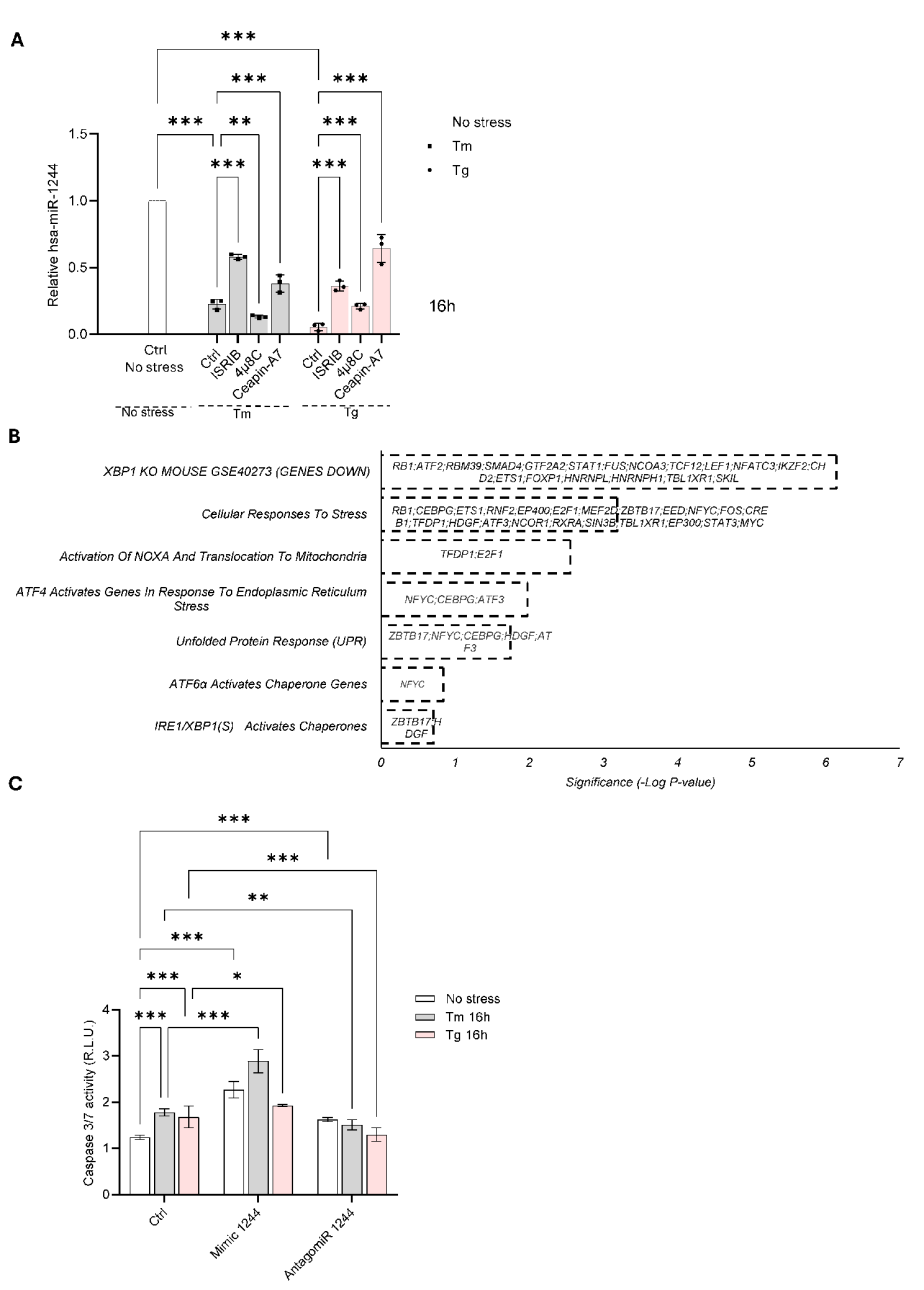
UPR pathway inhibitors affect miR-1244 levels during ER stress. (**A**) HeLa cells were treated with Tm (2.5 µg/ml) or Tg (500 nM) for 16 h with DMSO vehicle (Ctrl) or ISRIB (1 µM), 4µ8C (50µM), ceapin-A7(4µM), and total RNA enriched in miRNAs was isolated and miR-1244 levels were quantified by qPCR and normalized to *RNU48*. Data represents the mean ± SD of three experiments. *P < 0.05, **P < 0.001, ***P < 0.0001, were considered significant. (**B**) The summary of the gene ontology analysis of transcription factors binding sites in the pre-miR-1244 promoter region is shown. (**C**) HeLa cells were transfected with miR-1244 mimic, inhibitor or scramble control were exposed to ER stress for 16 h (Tm or Tg) and the caspase 3/7 activity was accessed and expressed as normalized relative light units were monitored every 15 min. The results from 6 measurements (*n* = 6) are plotted. Error bars represent standard deviations. *P* < 0.05, **P < 0.001, ***P < 0.0001 were considered significant

We also attempted to decipher the UPR mechanism responsible for miR-1244 reduction during ER stress. hsa-miR-1244 (also known as miR1244-1) is encoded on Chromosome 2: 231,713,314 − 231,713,398 forward strand (ENSG00000284378) and conserved only between *Homo sapiens* and *Pan troglodytes*. This genomic location is also occupied by prothymosin alpha, the *PTMA* gene (Chromosome 2: 231,706,895 − 231,713,551), but the *PTMA* expression remained constant during the ER stress (Supplemental Fig. 5). This suggests that downregulation of hsa-miR-1244 is independent of *PTMA*. Furthermore, we have not located an IRE1 consensus sequence within pre-miR-1244 and therefore excluded the possibility that this miRNA can be reduced via IRE1 associated degradation during ER stress. Therefore, to connect miR-1244 levels with the main arms of UPR, we have used inhibitors of the UPR branches that include 4µ8C for IRE1, ceapin-A7 for ATF6, and ISRIB that limits the PERK activities [33–35]. We applied these compounds during ER stress induced by Tm or Tg for 16 h. As shown in Fig. 3A, miR-1244 levels were increased in the presence of the ISRIB and ATF6 inhibitor, whereas the IRE1 inhibitor led to further reduction of this miRNA expression in Tm treated cells. A similar, but not identical pattern was observed in Tg treated cells, where ISRIB, ceapin-A7, and 4µ8C all rescued the miR-1244 levels. Taken together, the data pinpointed PERK and ATF6 signals as crucial for miR-1244 downregulation during ER stress and assigned IRE1 pathway with a modulatory role.

Next, we analyzed the promoter regions (chr2:231705255–231718193 (GRCh38/hg38)) for the UPR related factors using GeneHancer software [36]. This tested if the region was able to bind 336 transcription factors (TFs), among which a significant number were identified to be downregulated in mice with the *XBP1* knockout, while some of the others were specific for cellular stress responses (Fig. 3B). Interestingly, some of them were assigned to NOXA signaling, and that could explain why the expression of this gene was dramatically downregulated by both miR-1244 mimic and antagomiR (Fig. 3B). Notably, a group of UPR related transcription factors was identified as well and included TFs specific for all 3 main branches of these pathways: ATF6, IRE1 and PERK (ATF4) (Fig. 3B). Although these data do not define specific transcription factor responsible for miR-1244 expression during ER stress, they are in good agreement with the effects of UPR inhibitors and indicate that the crosstalk between the all main UPR branches determines the extend of miR-1244 reduction.

Since the miR-1244 target genes included both adaptive and apoptotic activities of UPR, to further investigate the impact of this miRNA on cell fate response to ER stress, we followed caspase 3 and 7 activities and HeLa cell viability using holomicroscopy based real time analysis [26] during UPR activation with both stressors and in the presence of miR-1244 analog and inhibitor.

As shown in Fig. 3C, after 16 h of exposure to both stressors, the caspase activity measured by quantifying the signal from the proluminescent caspase-3/7 DEVD-aminoluciferin substrate was moderately elevated, while miR-1244 overexpression increased the caspase activity in both stressed and nonstressed HeLa cells. Importantly, the relative luciferase signals observed during ER stress were comparable to previously reported in [18]. Notably, antagomiR transfection had a mild but significant reducing effect on ER stressed cells, while it increased significantly caspase activity in nonstress conditions to the levels observed in stress conditions.

**Fig. 4:**
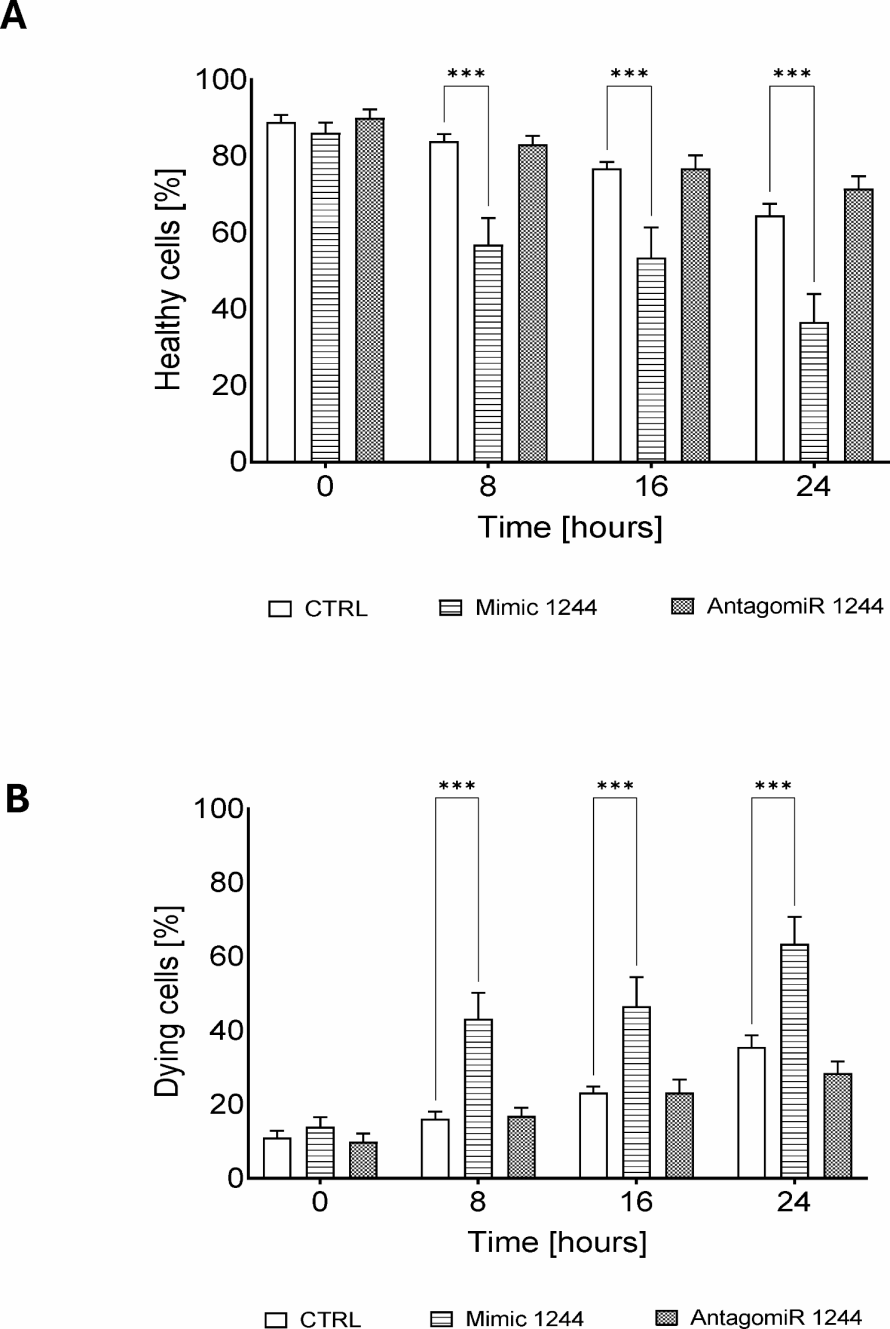
Overexpression of miR-1244 promotes cell death. The results of real-time monitoring of cell viability are shown with the real time and label free holographic microscopy using a HoloMonitor M4^®^ time-lapse cytometer of HeLa cells transfected with miR-1244 mimic or inhibitor or the scramble control and 48 h later monitored up to 24 h. Images were collected every 15 min (from 8 independent optical fields), and the distribution of live (blue) and dying cells (red) based on their optical thickness (Y-axis) and irregularity (X-axis) is presented at the 0, 8, 16 and 24 h time points. The images from up to 5 independent optical fields were collected and analyzed according to manufacturer’s instructions with HoloMonitor^®^ App Suite software. Representative samples are shown in Supplemental Fig. 6. For all analyses, the same cell parameter qualifications were applied. Experiments were performed in triplicate. Based on the cells irregularity and average optical thickness the percentages of healthy cells (**A**) and of dying cells (**B**) were calculated. Data represents the mean ± SE of three independent experiments. *P < 0.05, **P < 0.001, ***P < 0.0001 were considered significant

**Fig. 5:**
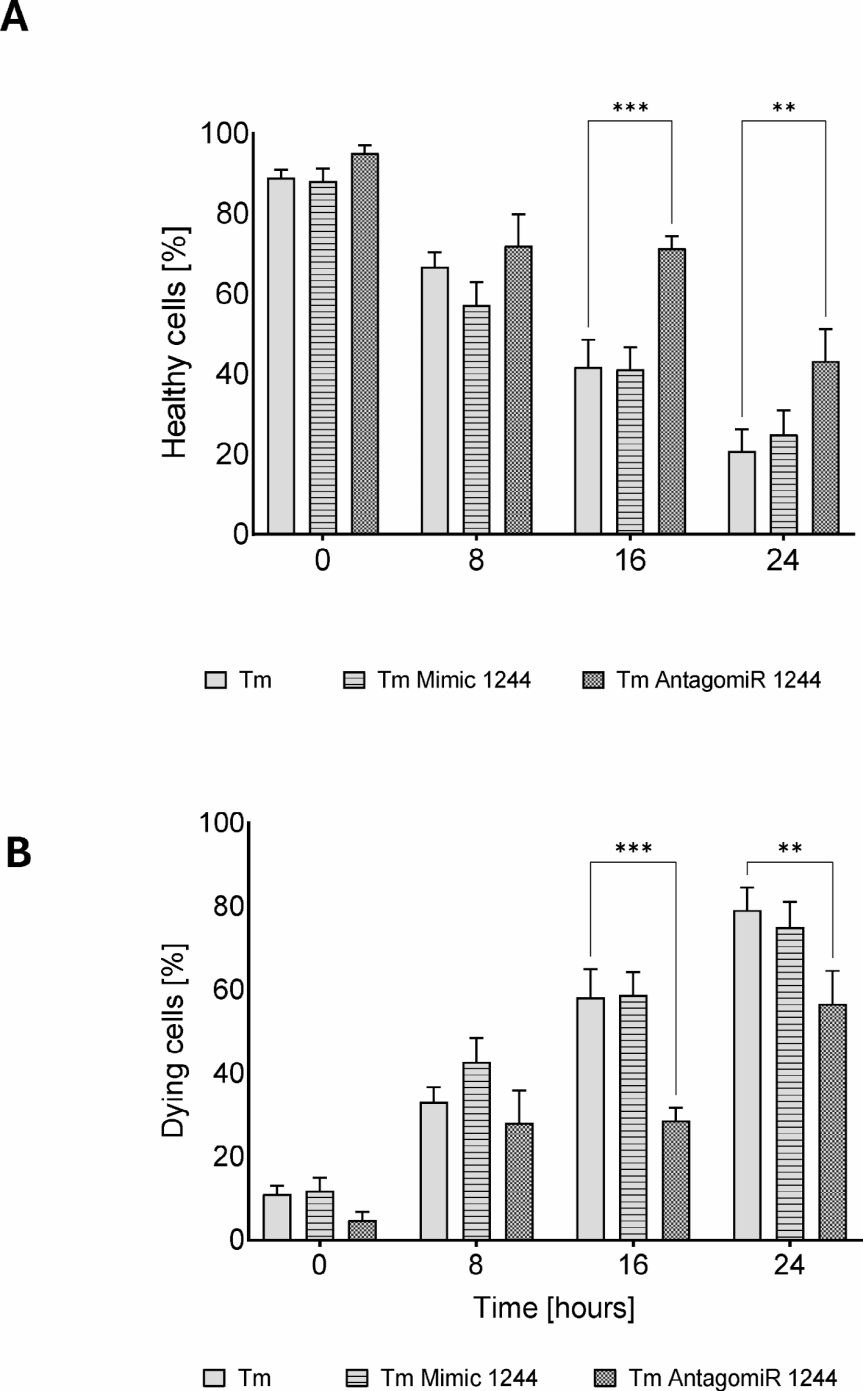
miR-1244 influences the fate of cells challenged with Tm induced ER stress. The results of real-time monitoring of cell viability with the real time and label free holographic microscopy are shown using a HoloMonitor M4^®^ time-lapse cytometer of HeLa cells transfected with miR-1244 mimic or inhibitor or the scramble control and 48 h later treated with Tm (2.5 µg/ml) up to 24 h. Images were collected every 15 min (from 5 independent optical fields), and the distribution of live (blue) and dying cells (red) based on their optical thickness (Y-axis) and irregularity (X-axis) is presented at the 0, 8, 16 and 24 h time points. The images from up to 5 independent optical fields were collected and analyzed according to manufacturer’s instructions with HoloMonitor^®^ App Suite software. Representative samples are shown in Supplemental Fig. 7. For all analyses, the same cell parameter qualifications were applied. Experiments were performed in triplicate. Based on the cells irregularity and average optical thickness the percentages of healthy cells (**A**) and of dying cells (**B**) were calculated. Data represents the mean ± SE of three independent experiments. *P < 0.05, **P < 0.001, ***P < 0.0001 were considered significant

**Fig. 6:**
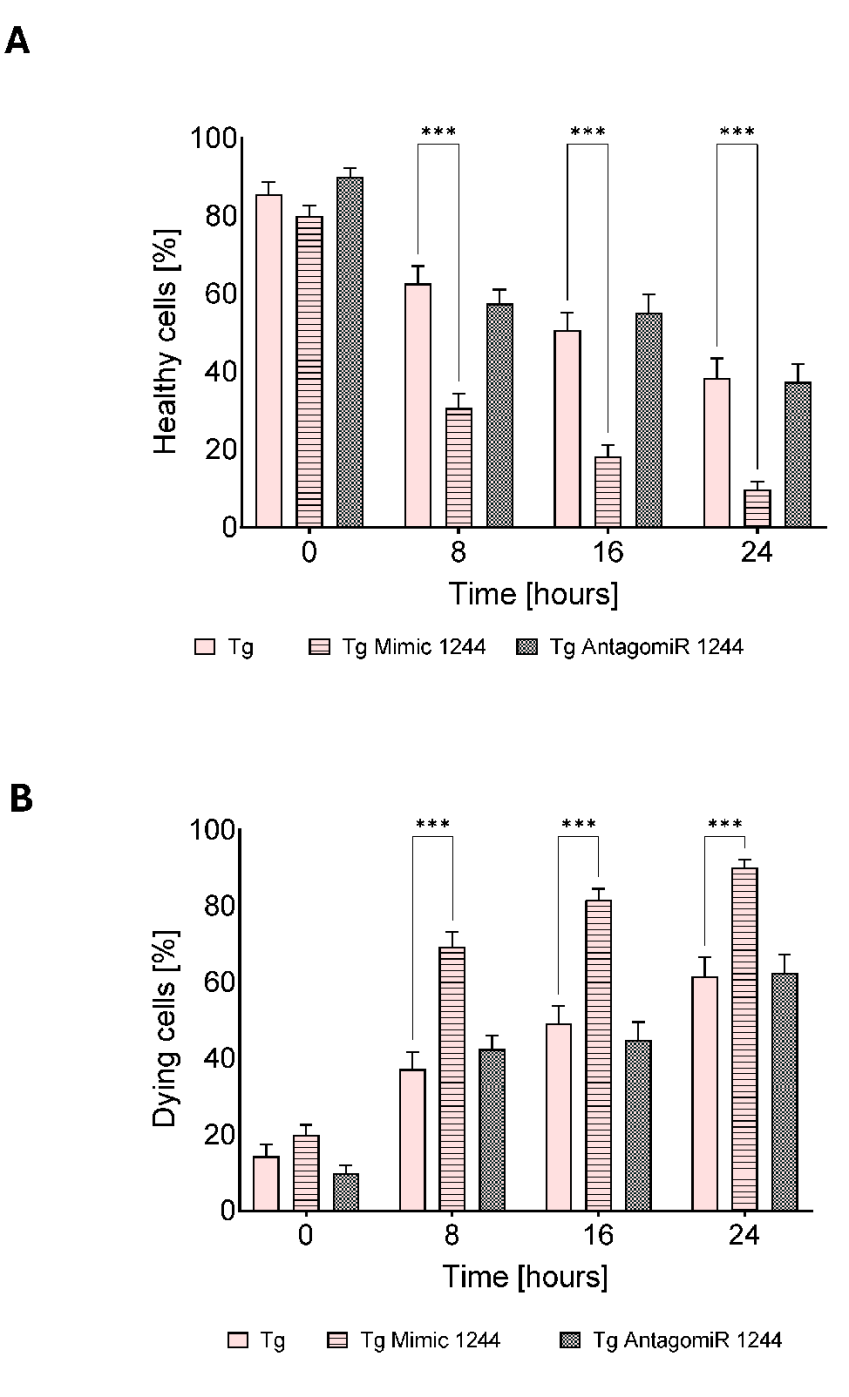
Exogenous miR-1244 influences the fate of cells challenged with Tg induced ER stress. The results of real-time monitoring of cell viability with the real time and label free holographic microscopy are shown using a HoloMonitor M4^®^ time-lapse cytometer of HeLa cells transfected with miR-1244 mimic or inhibitor or the scramble control and 48 h later treated with Tg (500 nM) up to 24 h. Images were collected every 15 min (from 5 independent optical fields), and the distribution of live (blue) and dying cells (red) based on their optical thickness (Y-axis) and irregularity (X-axis) is presented at the 0, 8, 16 and 24 h time points. The images from up to 5 independent optical fields were collected and analyzed according to manufacturer’s instructions with HoloMonitor^®^ App Suite software. Representative samples are shown in Supplemental Fig. 8. For all analyses, the same cell parameter qualifications were applied. Experiments were performed in triplicate. Based on the cells irregularity and average optical thickness the percentages of healthy cells (**A**) and of dying cells (**B**) were calculated. Data represents the mean ± SE of three independent experiments. *P < 0.05, **P < 0.001, ***P < 0.0001 were considered significant

To better understand this the cell fate decision dynamics, we performed real time and label free holographic microscopy-based monitoring of cell death and viability using a HoloMonitor^®^ time-lapse cytometer. Holographic microscopy was used to follow the optical thickness and irregularity of cells exposed for up to 24 h to Tm and Tg in the presence or absence of miR-1244 mimic or antagomiR (Figs. 4, 5 and 6 and Supplemental Figs. 6–8). Healthy cells are irregular in shape and thin, whereas dying cells are round and thick [25–28]. As shown in Fig. 4 and Supplemental Fig. 6, antagomiR had no significant effect on cellular survival in nonstress conditions, whereas after 8 h of experiment the miR-1244 mimic increased the number of dying cells even under nonstress conditions. In HeLa cells exposed to Tm, there was a significant increase in dying cells after 16 h of ER stress exposure, however, miR-1244 antagomir dramatically inhibited cell death after 16 and 24 h (Fig. 5, Supplemental Fig. 7). In Tg treated cells, the miR-1244 mimic significantly decreased the number of healthy cells starting at 8 h, and the antagomiR had similar levels of healthy cells as the Tg only treated cells, indicating that the antagomiR had no effect on cell survival during the Tg treatment (Fig. 6, Supplemental Fig. 8). In summary, the results suggest that ER stress reduction of miR-1244 expression contributes to the pro-survival arm of UPR.

## Discussion

The cell fate decisions during UPR are carefully balanced by both recovery and cell death arms of this complex pathway [12, 13]. This includes the consequences of ER stress altered microRNA expressions profiles as well as the involvement of a posttranscriptional modulation that remains poorly understood. Despite continuous research to identify the proadaptive and proapoptotic miRNAs involved in UPR, the main model remains selection of so-called “power switches”. These are the miRNAs that are dramatically induced by ER stress to downregulate single targets that are crucial to UPR to effectively modulate its output. However, the miRNA-based posttranscriptional regulation of cellular pathways is usually more complex and generally focuses on tuning multiple targets simultaneously via coordinated changes in many miRNA levels [37]. In contrast, each human transcript is modulated on average by at least four conserved miRNAs as well as many non-conserved ones [37, 38]. Furthermore, miRNAs can target hundreds of different mRNAs simultaneously in order to tune their expression and this makes these ncRNAs mild modulators of gene expression [39, 40]. Thus, despite identification of many miRNAs being induced by UPR and modulating the power switch function [15, 41–43], the question remains what is the functional relevance of miRNAs that are reduced during the UPR? Our previous studies clearly indicate that during ER stress, the global miRNA levels are downregulated [18].

In this study, our goal was to understand the relationship between the UPR and downregulation of miR-1244. We selected this specific miRNA in spite of the fact that it was poorly conserved. Our rationale was that miR-1244 was very unusual in that it was downregulated by ER stress in multiple cell lines as well as with five different pharmacological ER stressors. In fact, this was the only miRNA that fit these parameters. Furthermore, the role of miR-1244 in human physiology and pathology is poorly understood. Interestingly, there are reports that have correlated miR-1244 deregulation with schizophrenia [44], breast cancer [45], non-small lung cancer [46], ovarian cancer [47, 48] and osteosarcoma [49]. The proposed targets of miR-1244 were *serpine1* [50], centrosomal protein 55 (*CEP55*) [49] as well as MDM2 proto-oncogene (*MDM2*) [51]. However, none of these potential targets was affected either by ER stress or identified in our NGS mimic and inhibitor approaches. Thus, in our studies we focused on miRNA potential targets that were important for UPR and tried to address their changes with two independent ER stress models.

Although finding the exact miR-1244 target or targets that clearly define the demarcation between the apoptotic and adaptive arm of UPR is an important objective, it was technically challenging. Our speculation is that the complexity of the 3 pathways as well as the activation of both types of UPR responses of pro-survival and pro-apoptotic makes this a challenging endeavor. Furthermore, based on the analyses on the miR-1244 mimics and antagomiRs, miR-1244 appears to be acting indirectly on promoting apoptosis since we seldom saw bidirectional effects with mimic and antagomiR. Indeed, the differences in the expression profiles of miR-1244 between Tm- and Tg-treated cells are further supported by our previous observation that Tm-exposed cells are less prone to ER stress-related cell death than cells treated with Tg [6]. This phenomenon was observed starting from the 6th hour of treatment and became insignificant after 18 h [6]. Notably, in Tm-treated cells miR-1244 levels were reduced subsequently and rapidly after the treatment, whereas in Tg-treated cells this process was somewhat delayed. The fact that miR-1244 is reduced during the UPR highlights the fact that this is an important regulatory step to inhibit apoptosis and thereby promote cell survival during stress conditions.

Given that the different ER stress induction mechanisms modulate different effects on UPR signaling and the related cell fate decisions, it is interesting that a single miRNA can have such a profound effect on cell viability. Furthermore, determining the mechanism by which miR-1244 has this impact on cell viability during the UPR was extremely challenging. This is in part due to the fact that all the different pharmacological ER stress induction methods differ significantly in their effects on the transcriptional and postransciptional profiles of exposed cells. Consequently, finding concentrations and time frames for Tg and Tm treatments that result in the same transcriptomic and phenotypical effects is unlikely. Similar limitations apply to the crosstalk between all 3 UPR pathways that are simultaneously activated and modulated by different stressors. Consequently, this makes interpretation of data obtained with these inhibitors challenging. Finally, miRNA-based regulation relies on mild changes of a wide network of of different transcripts that include these miRNA-mRNAs ratios. Therefore, we did not expect to observe the same effects of miR-1244 reductions in Tm and Tg treated cells. We , however, believe that it is important not to draw conclusions based on only one pharmacological ER stress model.

That being said, our data strongly suggests that miR-1244 serves a complex modulatory role that has a potential link between the UPR and mitochondrial homeostasis as illustrated by miR-1244’s impact on *PUMA*, *NOXA*, and *HSPD1*. However, evaluating which of these potential miRNA targets are primary and which effects are secondary is difficult at best given the rather complex signaling crosstalk that occurs within the UPR. Thus, to fully understand the mode of action of this miR and identify its true targets, more precise tools are needed such as a clean reporter system that assesses the impact of endogenous miR-1244 on selected targets during the UPR. Furthermore, as shown by our experiments and analysis, the levels of miR-1244 are carefully controlled by PERK and ATF6 pathways, as well as modulated by IRE1. Thus, the loss of miR1244 is important for the pro-survival components of the UPR that is associated with all of the arms of the UPR.

Importantly, our studies are in good agreement with recent reports showing that the function of a microRNA can vary substantially and is dependent on its target gene expression levels [52]. Thus, depending on the pharmacological stressor type and to the UPR stage expression of its targets, miR-1244 changes may either be supporting these mRNAs reductions or accumulations. Furthermore, the levels of UPR related mRNAs being potential miR-1244 targets are rapidly and dramatically transcriptionally induced during UPR and the cellular miR-1244 levels may be not sufficient to stably sustain this regulatory function. However, further studies are needed to verify these points.

In summary, we identified novel a miRNA that integrates all of the arms of the UPR to modulate their activities and cell fate decisions. Although further studies are necessary to understand primary mechanism by which miR-1244 serves its pleiotropic modulatory function, we are the first to describe this complex mechanism by which this miRNA can serve as a regulatory hub during UPR and how elevated expression of it leads to pro-apoptotic responses.

## Electronic supplementary material

Below is the link to the electronic supplementary material.

**Supplemental Fig. 1**. (**A**). Relative levels of *HSPA5 (BIP)* and *DDIT3 (CHOP)* in total RNA samples from Calu 3 and 16HBE14o- cells subjected to ER stress with Tunicamycin (Tm) and ALLN are shown that were used for genome wide miRNA expression analysis. Calu 3 cells were exposed for 12 h to 5 µg/ml Tm or 100µM ALLN. 16HBE14o- cells were exposed for 9 h to 2.5 µg/ml Tm or 100µM ALLN. The data were collected from parallel genome wide mRNA expression analysis. (**B**) Furthermore, *HSPA5 (BIP)* and *DDIT3 (CHOP)* were quantified by qPCR and normalized to *GAPDH and 18S*. Data represents the mean ± SD of four experiments. **P < 0.001, ***P < 0.0001 were considered significant

**Supplemental Fig. 2**. The cytotoxic compound C-1305 does not affect levels of miR-1244. HeLa cells 16HBE14o- and cell were exposed to 10 µM C-1305 (IC_50_) for 24 h, and total RNA enriched in miRNAs was isolated and miR-1244 levels were quantified by qPCR and normalized to *RNU48*. Data represents the mean ± SD of three experiments

**Supplemental Fig. 3**. miR-1244 modulates expression of proadaptive UPR mediators. HeLa cells transfected with scramble control, miR-1244 mimic or antagomiR were treated with Tm (2.5 µg/ml) or Tg (500 nM) for 16 h and total RNA enriched in miRNAs was isolated. (**A**) *XBP1s*, (**B**) *ERN1*, (**C**) *PERK*, (**D**) *ATF6*, (**E**) *MAGT1*, (**F**) *EDEM1*, (**G**) *HSPD1*, and (**H**) *BIP* levels were quantified by qPCR and normalized to *SOD1*. Data represents the mean ± SD of three independent experiments. *P < 0.05, **P < 0.001, ***P < 0.0001 were considered significant

**Supplemental Fig. 4**. miR-1244 modulates expression of UPR related cell death mediators. HeLa cells transfected with scramble control, miR-1244 mimic or antagomiR were treated with Tm (2.5 µg/ml) or Tg (500 nM) for 16 h and total RNA enriched in miRNAs was isolated. (**A**) *BBC3*, (**B**) *NOXA*, (**C**) *XIAP*, (**D**) *CHOP* levels were quantified by qPCR and normalized to *SOD1*. Data represents the mean ± SD of three independent experiments. *P < 0.05, **P < 0.001, ***P < 0.0001 were considered significant

**Supplemental Fig. 5.** Relative levels of *PTMA* in total RNA samples 16HBE14o- cells subjected to ER stress with Tunicamycin (Tm) and ALLN are shown that were used for genome wide miRNA expression analysis. 16HBE14o- cells were exposed for 9 h to 2.5 µg/ml Tm or 100µM ALLN. The data were collected from parallel genome wide mRNA expression analysis. Data represents the mean ± SE of two experiments

**Supplemental Fig. 6**. Overexpression of miR-1244 promotes cell death. The results of real-time monitoring of cell viability are shown with the real time and label free holographic microscopy using a HoloMonitor M4^®^ time-lapse cytometer of HeLa cells transfected with miR-1244 mimic or inhibitor or the scramble control and 48 h later monitored up to 24 h. Images were collected every 15 min (from 8 independent optical fields), and the distribution of live (blue) and dying cells (red) based on their optical thickness (Y-axis) and irregularity (X-axis) is presented at the 0, 8, 16 and 24 h time points. The images from up to 5 independent optical fields were collected and analyzed according to manufacturer’s instructions with HoloMonitor^®^ App Suite software. Representative samples are shown. For all analyses, the same cell parameter qualifications were applied. Experiments were performed in triplicate

**Supplemental Fig. 7**. miR-1244 influences the fate of cells challenged with tm induced ER stress. The results of real-time monitoring of cell viability with the real time and label free holographic microscopy are shown using a HoloMonitor M4^®^ time-lapse cytometer of HeLa cells transfected with miR-1244 mimic or inhibitor or the scramble control and 48 h later treated with Tm (2.5 µg/ml) up to 24 h. Images were collected every 15 min (from 5 independent optical fields), and the distribution of live (blue) and dying cells (red) based on their optical thickness (Y-axis) and irregularity (X-axis) is presented at the 0, 8, 16 and 24 h time points. The images from up to 5 independent optical fields were collected and analyzed according to manufacturer’s instructions with HoloMonitor^®^ App Suite software. Representative samples are shown For all analyses, the same cell parameter qualifications were applied. Experiments were performed in triplicate

**Supplemental Fig. 8**. Exogenous miR-1244 influences the fate of cells challenged with tg induced ER stress. The results of real-time monitoring of cell viability with the real time and label free holographic microscopy are shown using a HoloMonitor M4^®^ time-lapse cytometer of HeLa cells transfected with miR-1244 mimic or inhibitor or the scramble control and 48 h later treated with Tg (500 nM) up to 24 h. Images were collected every 15 min (from 5 independent optical fields), and the distribution of live (blue) and dying cells (red) based on their optical thickness (Y-axis) and irregularity (X-axis) is presented at the 0, 8, 16 and 24 h time points. The images from up to 5 independent optical fields were collected and analyzed according to manufacturer’s instructions with HoloMonitor^®^ App Suite software. Representative samples are shown For all analyses, the same cell parameter qualifications were applied. Experiments were performed in triplicate

## Data Availability

All data generated or analyzed during this study are included in this published article (and its supplementary data set file). Deep sequencing data were deposited in Gene Expression Omnibus (GEO) at accession numbers: GSE117629 and GSE129813. The label-free holographic microscopy images were deposited at Zenodo database at 10.5281/zenodo.13359350.

## Abbreviations

16HBE14o: Immortalized human bronchial epithelial cells
ATF6: Activating transcription factor 6
BIP: Binding immunoglobulin protein
Calu-3: Human lung adenocarcinoma cell line
CHOP: CCAAT-enhancer-binding protein homologous protein
CEP55: Centrosomal protein 55
EDEM1: ER degradation enhancing alpha-mannosidase like protein 1
eIF2A: Eukaryotic initiation factor-2α
ER: Endoplasmic reticulum
ERAD: Endoplasmic reticulum-associated degradation
ERN1 a.k.a. IRE1: ER to nucleus signaling 1
GADD45A: Growth arrest and DNA damage inducible alpha
HeLa: Human cervix adenocarcinoma cells
Hsp60: Heat shock protein family D
HSPD1: Member 1
IRE-1: Inositol-requiring enzyme-1
JNK: c-Jun N-terminal kinase
MAGT1: Magnesium transporter 1
MDM2: MDM2 proto-oncogene, E3 ubiquitin protein ligase
miRNA: microRNA
PERK: PKR-like endoplasmic reticulum kinase
PTMA: Prothymosin alpha
PUMA, a.k.a. BBC3: BCL2-binding component 3
PMAIP, a.k.a. NOXA: Phorbol-12-myristate-13-acetate-induced protein 1
RIDD: Regulated IRE-1α-dependent decay
SOD1: Superoxide dismutase 1
UPR: Unfolded protein response
XBP-1s: Spliced X-box-binding protein 1
XPR1: Xenotropic and polytropic retrovirus receptor 1
XIAP: X-linked inhibitor of apoptosis
