# Supplementary figures and images for: The Yin and Yang of hsa-miR-1244 expression levels during activation of the UPR control cell fate
Source: Cell Commun Signal. 2024 Dec 2;22:577. doi: 10.1186/s12964-024-01967-2 (PMC11610070; doi:10.1186/s12964-024-01967-2)

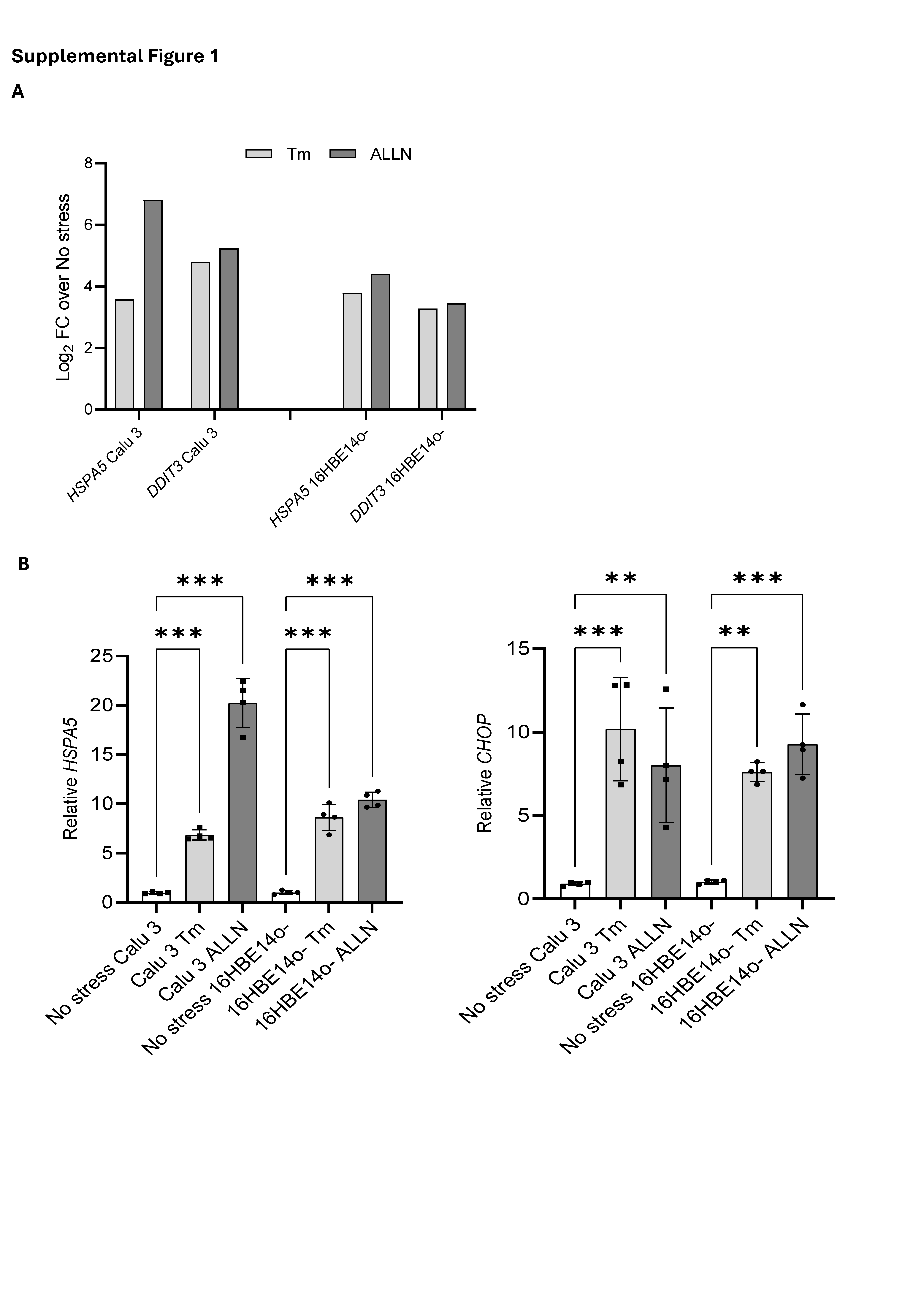

Supplement: Supplementary file 1 — Supplemental Fig. 1. (A). Relative levels of HSPA5 (BIP) and DDIT3 (CHOP) in total RNA samples from Calu 3 and 16HBE14o- cells subjected to ER stress with Tunicamycin (Tm) and ALLN are shown that were used for genome wide miRNA expression analysis. Calu 3 cells were exposed for 12 h to 5 µg/ml Tm or 100µM ALLN. 16HBE14o- cells were exposed for 9 h to 2.5 µg/ml Tm or 100µM ALLN. The data were collected from parallel genome wide mRNA expression analysis. (B) Furthermore, HSPA5 (BIP) and DDIT3 (CHOP) were quantified by qPCR and normalized to GAPDH and 18S. Data represents the mean ± SD of four experiments. **P < 0.001, ***P < 0.0001 were considered significant [file 12964_2024_1967_MOESM1_ESM.tif]

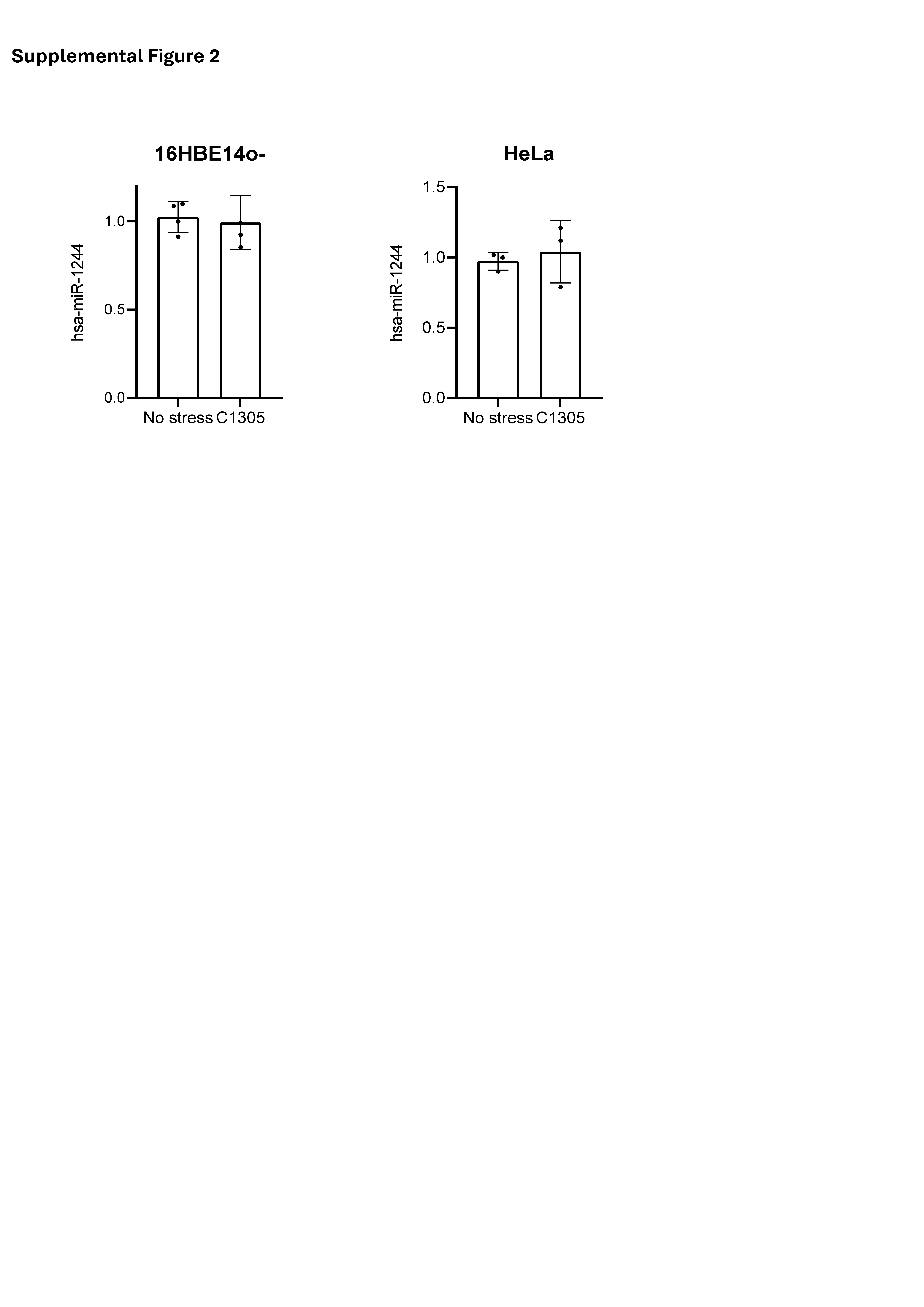

Supplement: Supplementary file 2 — Supplemental Fig. 2. The cytotoxic compound C-1305 does not affect levels of miR-1244. HeLa cells 16HBE14o- and cell were exposed to 10 µM C-1305 (IC50) for 24 h, and total RNA enriched in miRNAs was isolated and miR-1244 levels were quantified by qPCR and normalized to RNU48. Data represents the mean ± SD of three experiments [file 12964_2024_1967_MOESM2_ESM.tif]

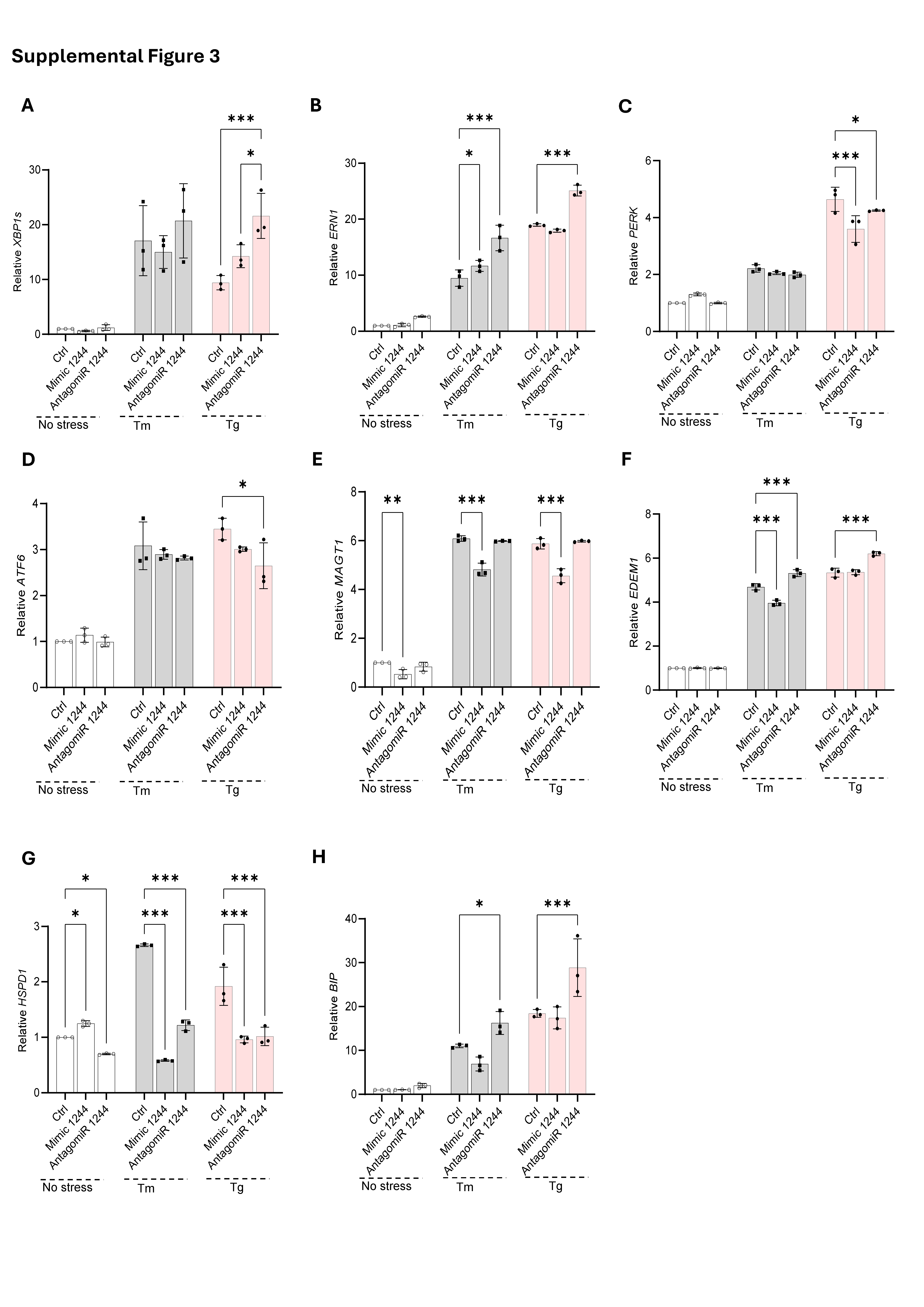

Supplement: Supplementary file 3 — Supplemental Fig. 3. miR-1244 modulates expression of proadaptive UPR mediators. HeLa cells transfected with scramble control, miR-1244 mimic or antagomiR were treated with Tm (2.5 µg/ml) or Tg (500 nM) for 16 h and total RNA enriched in miRNAs was isolated. (A) XBP1s, (B) ERN1, (C) PERK, (D) ATF6, (E) MAGT1, (F) EDEM1, (G) HSPD1, and (H) BIP levels were quantified by qPCR and normalized to SOD1. Data represents the mean ± SD of three independent experiments. *P < 0.05, **P < 0.001, ***P < 0.0001 were considered significant [file 12964_2024_1967_MOESM3_ESM.tif]

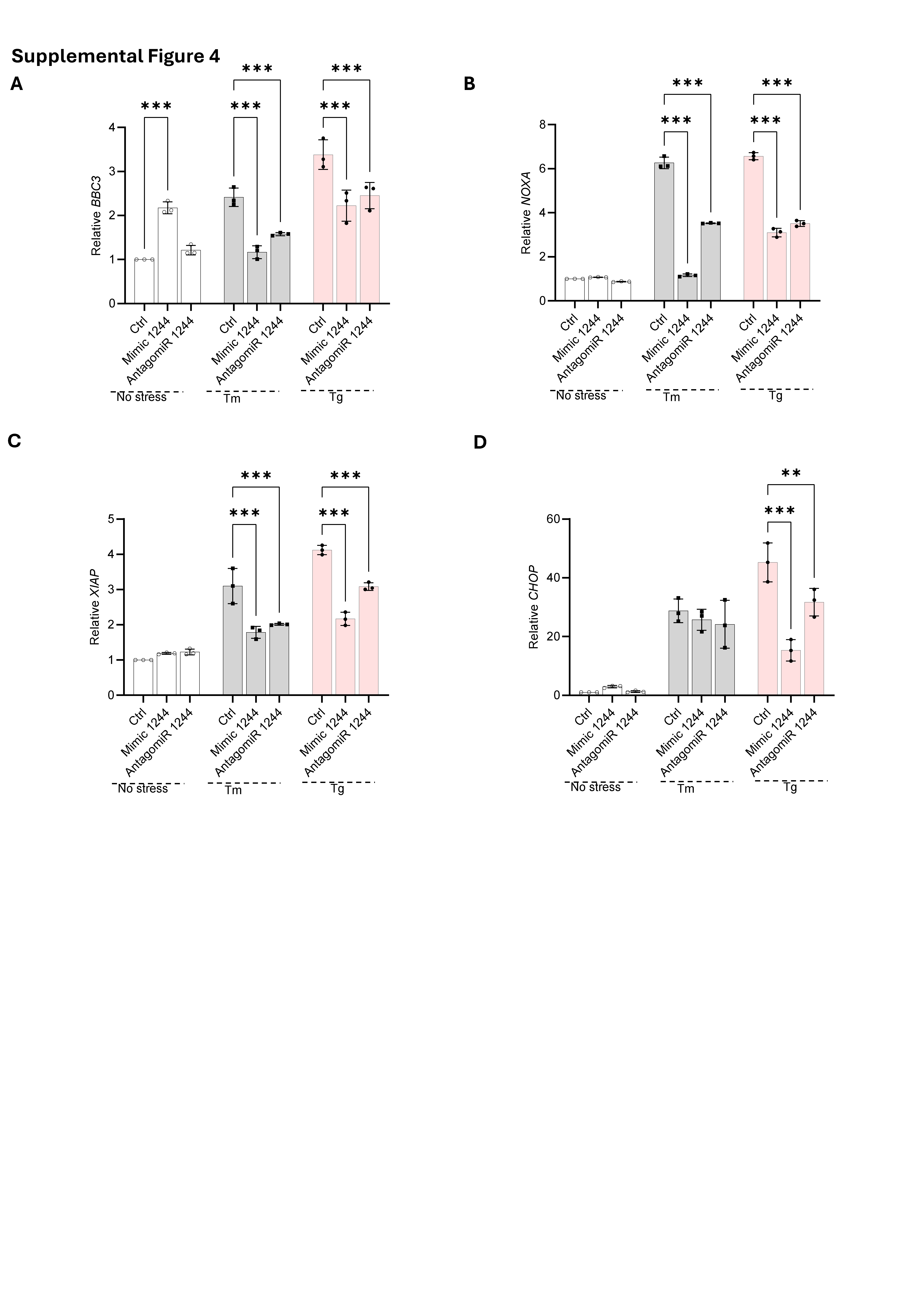

Supplement: Supplementary file 4 — Supplemental Fig. 4. miR-1244 modulates expression of UPR related cell death mediators. HeLa cells transfected with scramble control, miR-1244 mimic or antagomiR were treated with Tm (2.5 µg/ml) or Tg (500 nM) for 16 h and total RNA enriched in miRNAs was isolated. (A) BBC3, (B) NOXA, (C) XIAP, (D) CHOP levels were quantified by qPCR and normalized to SOD1. Data represents the mean ± SD of three independent experiments. *P < 0.05, **P < 0.001, ***P < 0.0001 were considered significant [file 12964_2024_1967_MOESM4_ESM.tif]

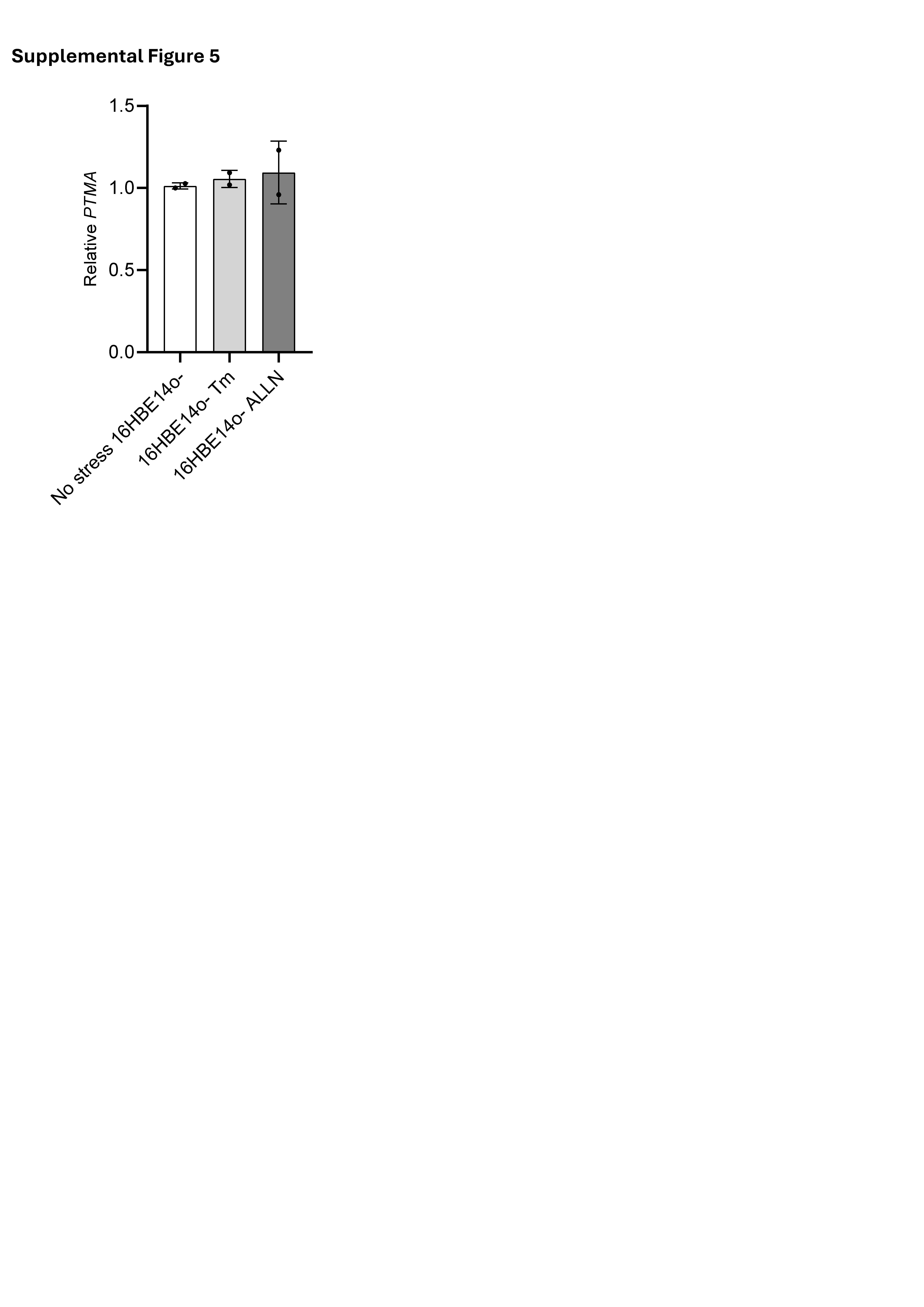

Supplement: Supplementary file 5 — Supplemental Fig. 5. Relative levels of PTMA in total RNA samples 16HBE14o- cells subjected to ER stress with Tunicamycin (Tm) and ALLN are shown that were used for genome wide miRNA expression analysis. 16HBE14o- cells were exposed for 9 h to 2.5 µg/ml Tm or 100µM ALLN. The data were collected from parallel genome wide mRNA expression analysis. Data represents the mean ± SE of two experiments [file 12964_2024_1967_MOESM5_ESM.tif]

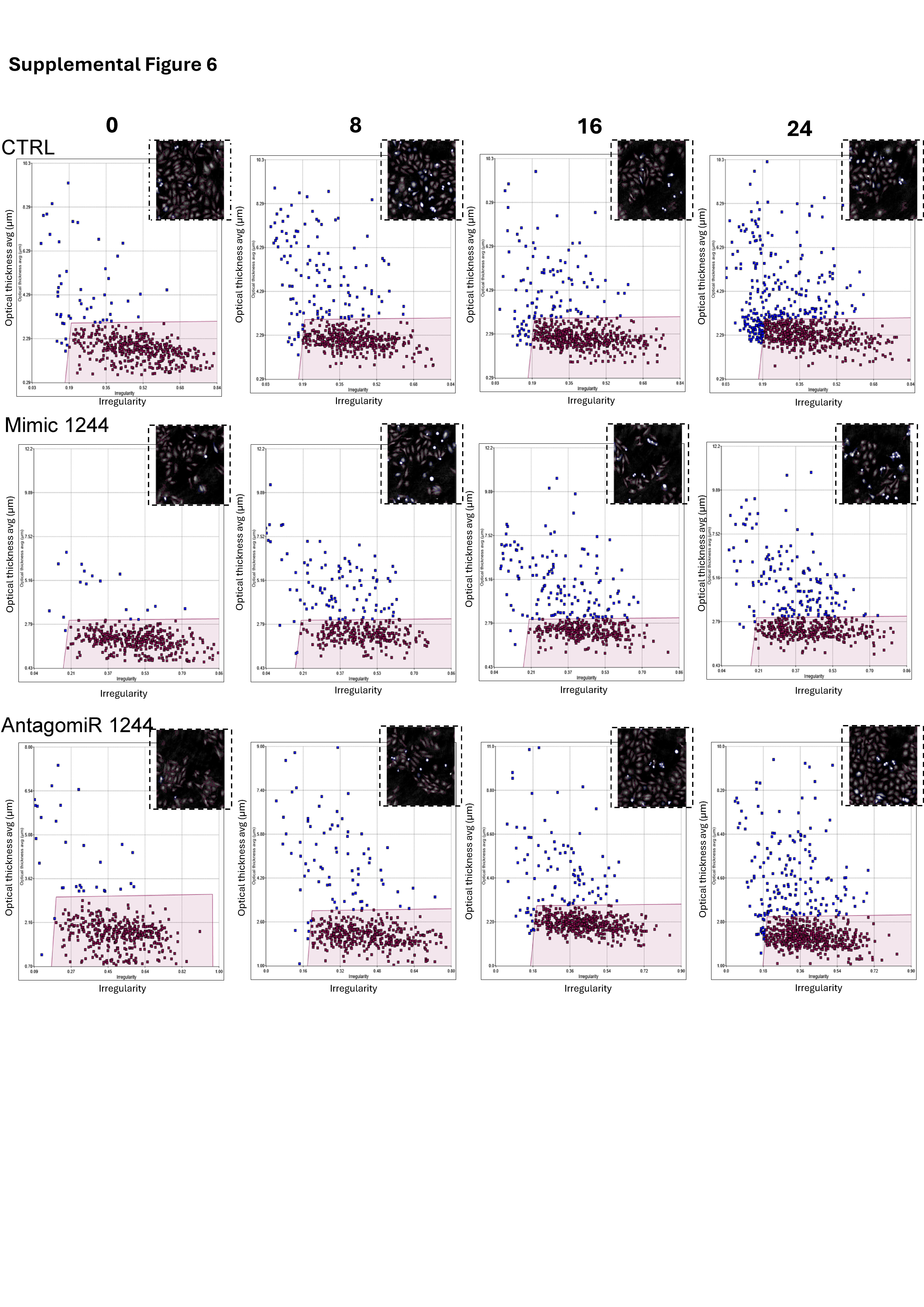

Supplement: Supplementary file 6 — Supplemental Fig. 6. Overexpression of miR-1244 promotes cell death. The results of real-time monitoring of cell viability are shown with the real time and label free holographic microscopy using a HoloMonitor M4® time-lapse cytometer of HeLa cells transfected with miR-1244 mimic or inhibitor or the scramble control and 48 h later monitored up to 24 h. Images were collected every 15 min (from 8 independent optical fields), and the distribution of live (blue) and dying cells (red) based on their optical thickness (Y-axis) and irregularity (X-axis) is presented at the 0, 8, 16 and 24 h time points. The images from up to 5 independent optical fields were collected and analyzed according to manufacturer’s instructions with HoloMonitor® App Suite software. Representative samples are shown. For all analyses, the same cell parameter qualifications were applied. Experiments were performed in triplicate [file 12964_2024_1967_MOESM6_ESM.tif]

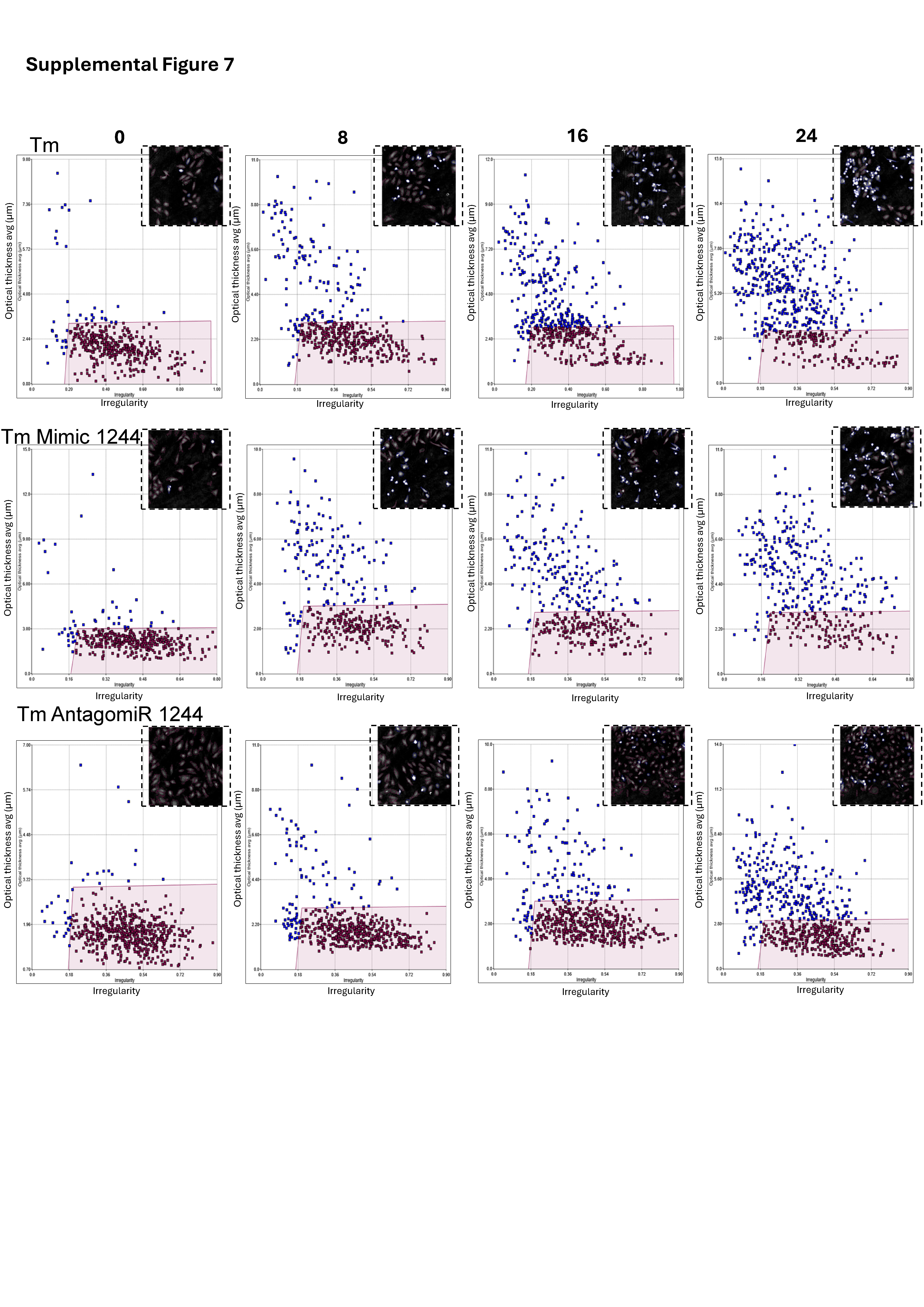

Supplement: Supplementary file 7 — Supplemental Fig. 7. miR-1244 influences the fate of cells challenged with tm induced ER stress. The results of real-time monitoring of cell viability with the real time and label free holographic microscopy are shown using a HoloMonitor M4® time-lapse cytometer of HeLa cells transfected with miR-1244 mimic or inhibitor or the scramble control and 48 h later treated with Tm (2.5 µg/ml) up to 24 h. Images were collected every 15 min (from 5 independent optical fields), and the distribution of live (blue) and dying cells (red) based on their optical thickness (Y-axis) and irregularity (X-axis) is presented at the 0, 8, 16 and 24 h time points. The images from up to 5 independent optical fields were collected and analyzed according to manufacturer’s instructions with HoloMonitor® App Suite software. Representative samples are shown For all analyses, the same cell parameter qualifications were applied. Experiments were performed in triplicate [file 12964_2024_1967_MOESM7_ESM.tif]

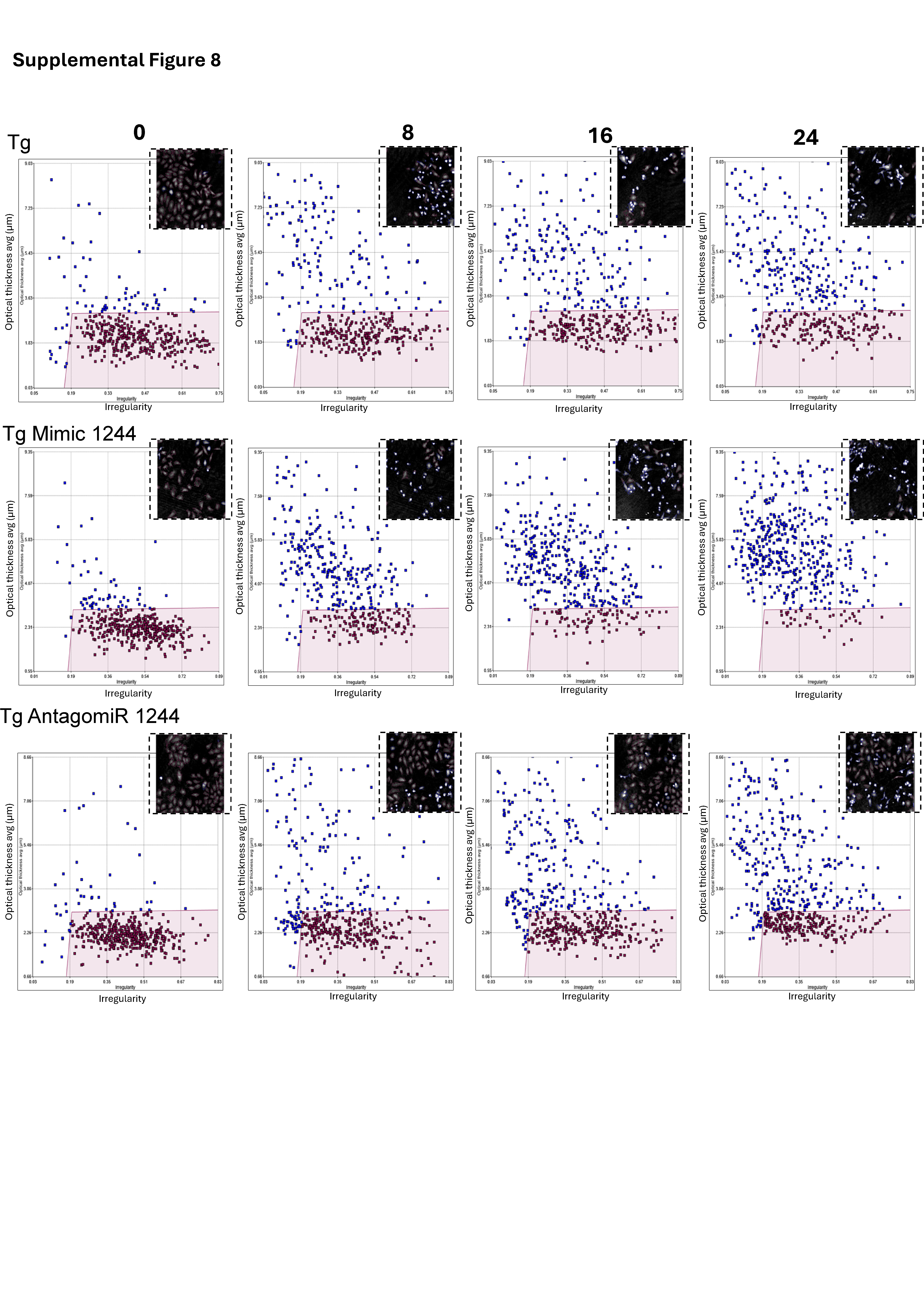

Supplement: Supplementary file 8 — Supplemental Fig. 8. Exogenous miR-1244 influences the fate of cells challenged with tg induced ER stress. The results of real-time monitoring of cell viability with the real time and label free holographic microscopy are shown using a HoloMonitor M4® time-lapse cytometer of HeLa cells transfected with miR-1244 mimic or inhibitor or the scramble control and 48 h later treated with Tg (500 nM) up to 24 h. Images were collected every 15 min (from 5 independent optical fields), and the distribution of live (blue) and dying cells (red) based on their optical thickness (Y-axis) and irregularity (X-axis) is presented at the 0, 8, 16 and 24 h time points. The images from up to 5 independent optical fields were collected and analyzed according to manufacturer’s instructions with HoloMonitor® App Suite software. Representative samples are shown For all analyses, the same cell parameter qualifications were applied. Experiments were performed in triplicate [file 12964_2024_1967_MOESM8_ESM.tif]
